# Multivariate pattern analysis: a method and software to reveal, quantify, and visualize predictive association patterns in multicollinear data

**DOI:** 10.1186/s12859-024-05660-6

**Published:** 2024-01-31

**Authors:** Tim U. H. Baumeister, Eivind Aadland, Roger G. Linington, Olav M. Kvalheim

**Affiliations:** 1 Department of Chemistry, Simon Fraser University, Burnaby, Canada; 2 Department of Sport, Food and Natural Sciences, Western Norway University of Applied Sciences, Sogndal, Norway; 3 Department of Chemistry, University of Bergen, Bergen, Norway

**Keywords:** Multivariate pattern analysis, Multicollinear covariates, Net association patterns, Latent variable projection, Covariate projection, Target projection

## Abstract

**Background:**

Strongly multicollinear covariates, such as those typically represented in metabolomics applications, represent a challenge for multivariate regression analysis. These challenges are commonly circumvented by reducing the number of covariates to a subset of linearly independent variables, but this strategy may lead to loss of resolution and thus produce models with poorer interpretative potential. The aim of this work was to implement and illustrate a method, multivariate pattern analysis (MVPA), which can handle multivariate covariates without compromising resolution or model quality.

**Results:**

MVPA has been implemented in an open-source R package of the same name, mvpa. To facilitate the usage and interpretation of complex association patterns, mvpa has also been integrated into an R shiny app, mvpaShiny, which can be accessed on www.mvpashiny.org. MVPA utilizes a general projection algorithm that embraces a diversity of possible models. The method handles multicollinear and even linear dependent covariates. MVPA separates the variance in the data into orthogonal parts within the frame of a single joint model: one part describing the relations between covariates, outcome, and explanatory variables and another part describing the “net” predictive association pattern between outcome and explanatory variables. These patterns are visualized and interpreted in variance plots and plots for pattern analysis and ranking according to variable importance. Adjustment for a linear dependent covariate is performed in three steps. First, partial least squares regression with repeated Monte Carlo resampling is used to determine the number of predictive PLS components for a model relating the covariate to the outcome. Second, postprocessing of this PLS model by target projection provided a single component expressing the predictive association pattern between the outcome and the covariate. Third, the outcome and the explanatory variables were adjusted for the covariate by using the target score in the projection algorithm to obtain “net” data. We illustrate the main features of MVPA by investigating the partial mediation of a linearly dependent metabolomics descriptor on the association pattern between a measure of insulin resistance and lifestyle-related factors.

**Conclusions:**

Our method and implementation in R extend the range of possible analyses and visualizations that can be performed for complex multivariate data structures. The R packages are available on github.com/liningtonlab/mvpa and github.com/liningtonlab/mvpaShiny.

**Supplementary Information:**

The online version contains supplementary material available at 10.1186/s12859-024-05660-6.

## Introduction

Data are increasingly multivariate and collinear within most application areas. This has caused a steady increase in the use of latent variable projection (LVP) methods for data analysis and modeling [1] and references therein. LVP methods provide association patterns as linear combinations of the measured variables. They share a common mathematical basis, but the appropriate methods to use are selected according to criteria relevant for the problem at hand. Principal component analysis (PCA) [2] for data exploration and partial least squares (PLS) for regression modeling [3] are LVP methods that are currently used on a routine basis. These and other available methods can be described within a general projection algorithm [4], which has been expanded with criteria facilitating interpretation and visualization of models [5] and references therein.

Multivariate pattern analysis (MVPA) [6, 7] is a variant of latent variable regression (LVR) focusing on interpretation and visualization of collinear data in terms of *predictive* association patterns. The key steps of MVPA are as follows: (i) Quantify, visualize, and adjust for the influence of covariates on the outcome and the explanatory variables. (ii) Use PLS regression with repeated Monte Carlo resampling [8] to obtain a *predictive* model between the adjusted (net) outcome and explanatory variables. (iii) Postprocess the PLS model by performing a target projection (TP) [5, 9] to obtain the predictive association pattern of the explanatory variables to the outcome. (iv) Calculate measures of variable importance, e.g., selectivity ratio [10], to quantify and visualize the net association patterns.

When using multiple linear regression (MLR), explanatory variables (including covariates) are traditionally mutually adjusted by their inclusion in a joint statistical model, given that this model allows for interpretation of the explanatory variables’ independent associations with the outcome. However, this procedure is not suited for multicollinear descriptors where associations are *not* independent but collinear and even linearly dependent. When the mutual correlations of covariates are relatively weak, the influence of covariates can be eliminated by regressing either the explanatory variables or the outcome (or both) on the covariates using MLR and applying the residuals from these models in further analysis [7]. However, when the correlations are strong, this is no longer a suitable approach. To solve this problem, we used principal components to adjust for even linearly dependent covariates and developed tools to quantify and visualize the influence of covariates on association patterns by, e.g., variance plots [11, 12]. However, many principal components are usually needed to represent the covariates, which is a drawback for interpretation and visualization. Thus, we recently refined this procedure by regressing linear dependent covariates on the outcome using PLS and then postprocessing by target projection to obtain the predictive association pattern of the covariates to the outcome [13]. The score on the target component, for each multivariate covariate, was subsequently used in a covariate projection to adjust both outcome and explanatory variables for the covariates prior to further modelling.

MVPA is a general tool for modeling, interpretation, and visualization of association patterns in the presence of covariates. In the next section, we describe the projection algorithm, how to handle univariate and multivariate covariates using this algorithm, and the main features of our MVPA software. We then apply the software to quantify how a linearly dependent metabolomics covariate partially mediates the association between lifestyle-related factors and a measure of insulin resistance. This application aims to show the data-analytical steps and some important visualization tools that are available in the developed software.

## Methods, algorithms, and software

This section summarizes the key elements of our approach and the software.

### Problem specification

The aim is to model the net association pattern between an outcome *y* and *M* explanatory variables, {*x*_*1*_, *x*_*2*_, …, *x*_*M*_}, in the presence of *K* variables, {*z*_*1*_, *z*_*2*_, …, *z*_*K*_}, covarying with the outcome and the explanatory variables. The net association pattern is defined as the pattern obtained after removal of the influence of covariates. The vector ***y*** contains the measurements for *y* and the matrices ***X*** and ***Z***, the corresponding measurements for the *x*- and *z*-variables, respectively. The covariates can, for instance, be confounders or mediators, as in the application we use to illustrate the approach and the software below. Covariates can be univariate or multivariate. In the application studied here, we have both univariate and multivariate covariates.

The net vector ***y***_*net*_ and net matrix ***X***_*net*_ for the outcome and explanatory variables, respectively, are defined as:

1a $${{\varvec{y}}}_{net }={\varvec{y}}- {\widehat{{\varvec{y}}}}_{cov}$$

1b $${{\varvec{X}}}_{net} ={\varvec{X}}- {\widehat{{\varvec{X}}}}_{cov}$$

Here, ${\widehat{{\varvec{y}}}}_{cov}$ and ${\widehat{{\varvec{X}}}}_{cov}$ represent the part of ***y*** and ***X*** explained when regressing them on the covariates using the projection methods discussed in the next sections.

The vector of net regression coefficients ***b***_*net*_ is subsequently derived from the regression model

2 $${{\varvec{y}}}_{net}= {{\varvec{X}}}_{net}{{\varvec{b}}}_{net}+ {{\varvec{e}}}_{y}$$

Equations (1a), (1b), and (2) imply the following decompositions of ***y*** and ***X***:

3a $${\varvec{y}} = {\widehat{{\varvec{y}}}}_{cov}+ {\widehat{{\varvec{y}}}}_{net}+ {{\varvec{e}}}_{y}$$

3b $${\varvec{X}}= {\widehat{{\varvec{X}}}}_{cov}+ {\widehat{{\varvec{X}}}}_{net}+ {{\varvec{E}}}_{X}$$

where ${{\varvec{e}}}_{y}$ and ${{\varvec{E}}}_{X}$ correspond to the residual vector and matrix for ***y*** and ***X***, respectively, after accounting for the predictive parts of the covariates and the outcome and the explanatory variables.

### Latent-variable projection methods

LVP methods decompose multicollinear data into linear combinations of the measured variables according to criteria adapted to solve the problem at hand. The projection algorithm [4, 5] consists of four steps:

For *a* = *1*,*2*, …, *A*

1. Select a weight vector ***w***_*a*_ normalized to unit length, i.e., ‖***w***_*a*_‖ = *1*.
2. Calculate the score vector ***t***_*a*_ as ***t***_*a*_ = ***X***_*a*_***w***_*a*_.
3. Calculate the loading vector ***p***_*a*_ as ***p***_*a*_ = ***X***_*a*_^*T*^***t***_*a*_/(***t***_*a*_^*T*^***t***_*a*_).
4. Remove dimension a from ***X***_*a*_ by subtracting the product of the score and loading vector, ***X***_*a*+*1*_ = ***X***_***a***_ − ***t***_*a*_***p***_*a*_^*T*^.

*A* is the total number of latent variables extracted.

The algorithm is initialized with ***X***_*1*_ = ***X***, i.e., the column-centered (and pretreated) matrix ***X***.

Initiate the algorithm with a randomly chosen weight vector and iterate between 2 and 3 until ***p***_*1*_/‖***p***_*1*_‖ = ***w***_*1*_. This uniquely defines the first principal component. After the orthogonalization in step 4, the second principal component can be extracted in the same way. The process continues until the *A* components have been calculated.

**Fig. 1:**
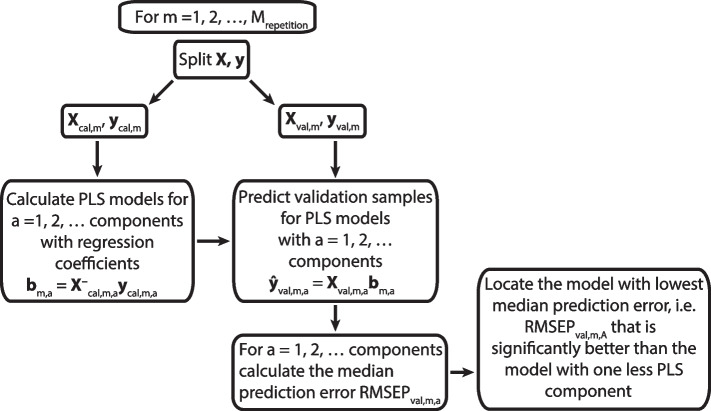
Flow diagram of the repeated Monte Carlo resampling algorithm used to validate the PLS models. This automatic procedure only needs three user defined inputs: **i** the number of repetitions, **ii** the maximum number of components in PLS models, and, **iii** the fraction of the distribution of the root mean squared prediction errors (RMSEPs) for the PLS model with lowest median that is higher than the median RMSEP for the model with one PLS component less. This fraction must be lower than 0.5 in order to protect against overfitting

Using the normalized vector of covariances between the explanatory variables and the outcome as a weight vector in step 1, i.e., ***w***_*a*_ = ***X***_*a*_^*T*^***y***/‖***X***_*a*_^*T*^***y***‖, provides the PLS solution. The number of PLS components is determined by optimizing the predictive ability of the model. Several procedures have been developed for this purpose. MVPA uses a procedure based on repeated Monte Carlo resampling [8]. Figure 1 describes the algorithm used in this work.

Other choices for projections are available. By means of target projection (TP) [5, 9, 14], a single *predictive* latent variable is produced quantifying the association pattern between the outcome and the explanatory variables. In our MVPA implementation, the target component is derived from a validated PLS model by choosing the normalized regression vector ***b*** for the PLS model as the weight vector, i.e., ***w***_*TP*_ = ***b***/‖***b***‖ in the projection algorithm above, but the regression vector from other regression techniques can be used as well. Target projection provides a single pair of score and loading vectors, denoted ***t***_*TP*_ and ***p***_*TP*_, respectively, which optimally describe the association pattern of the *x*-variables with the predicted *y*-variable. Thus, the projection algorithm is used twice: first, to derive a validated PLS model, and second, to calculate a single predictive latent variable expressing the *predictive* association pattern. Below, we show how the same targeted approach can be used to handle multivariate covariates.

### Projections to quantify and isolate the influence of covariates

The aims of our approach to handle covariates are two-fold: (i) determine how covariates influence the predictive association pattern between the outcome variable and the explanatory variables, and (ii) remove the influence of the covariates on the outcome and explanatory variables to determine the net (independent) association pattern of the explanatory variables to the outcome. Covariates are often present as confounders in statistical analysis, but covariates may also appear in a broader and more complex context, as in our application below.

Consider first the case of a single univariate covariate. By using the projection algorithm above, it is possible to eliminate the influence of the covariate on both the outcome ***y*** and the explanatory variables ***X*** simultaneously by simply augmenting the column-centred matrix ***X*** with one extra column for the centred outcome ***y*** and one for the centred covariate ***z*** to create the matrix ***X***_*aug,1*_ = [***y z X***]. We define the corresponding covariate projection (CP) through the weight vector ***w***_*CP*_ with all elements equal to zero except the element corresponding to the position of the covariate in ***X***_*aug,1*_ and carry out steps 2–4 in the projection algorithm above.

The column in the residual matrix ***X***_*aug,2*_ corresponding to the outcome variable is then ***y***_*net*_ = ***y*** − ***y(z***^*T*^***y)***/(***z***^*T*^***z***), which is the residual of ***y*** obtained by regressing the outcome on the covariate. Similarly, the residual vectors of the *x*-variables after CP on the covariate are ***x***_*net,i*_ = ***x***_i_ − ***x***_*i*_(***z***^*T*^***x***_*i*_)/(***z***^*T*^***z***) for *i* = *1*,*2*, …,*M*. The column in ***X***_*aug*_*,*_*2*_ representing the residuals of the covariate after CP is a vector where all elements are zero, i.e., ***e***_*z*_ = ***z*** − ***z***(***z***^*T*^***z***)/(***z***^*T*^***z***) = ***0***. Thus, for a single covariate, the residual matrix ***X***_*aug,2*_ contains the adjusted outcome and explanatory variables and a column of zeros for the covariate.

Generalization to *A* covariates, not being multicollinear, is straightforward: Augment ***X*** by one column for each covariate to produce the matrix ***X***_*aug,1*_ = [***y Z X***]. Then, perform the CP procedure as many times as there are covariates. The elements in the residual matrix ***X***_*aug,A*+*1*_ are zero for all the covariates.

### Strategy to handle multicollinear covariates

Adjustments can be performed stepwise for each covariate using the projection algorithm as shown in the previous section, but adjustment of outcome and explanatory variables to obtain net data can also be achieved by calculating regression models including all the covariates simultaneously:

4a $${\varvec{y}}= {{\varvec{Z}}{\varvec{b}}}_{Z,y}+ {{\varvec{y}}}_{net}$$

4b $${\varvec{x}}_{i} = {\varvec{Zb}}_{{Z,x_{i} }} + {\varvec{x}}_{net,i} \;\;\{ i = 1,2, \ldots ,M\}$$

The residuals ***y***_*net*_ and {***x***_*net,i*_, *i* = *1*, *2*, …, *M*} are subsequently used in Eq. 2 to calculate the net regression coefficients associated with the outcome and the explanatory variables.

Assuming that the matrix **Z** is of full rank, MLR can be used to calculate the regression vectors from Eqs. (4a) and (4b). However, multivariate covariates may be linearly dependent, implying that the assumption of full rank fails. In such cases, PLS can be used to solve Eqs. 4a and 4b by calculating a Moore–Penrose pseudoinverse [15] to establish the relation of the covariates to the outcome and the explanatory variables. However, a drawback is the interpretation and visualization of models since more than one PLS component is usually needed to represent a multivariate covariate. However, this can be circumvented by using target projection as a postprocessing step.

In the case of linear dependency among covariates, we cannot use the projection algorithm for the covariates directly. For such cases, we first use PLS to model the relation between the outcome and the multivariate covariate, then use the projection algorithm to obtain a single predictive target component and, finally, adjust for the multivariate covariate in a covariate projection using the score vector for the target component [13]. This procedure retains the option of using the projection algorithm above stepwise and thus relating single or groups of covariates to their specific association patterns with outcome and exploratory variables, providing enhanced possibilities for interpretation.

**Fig. 2:**
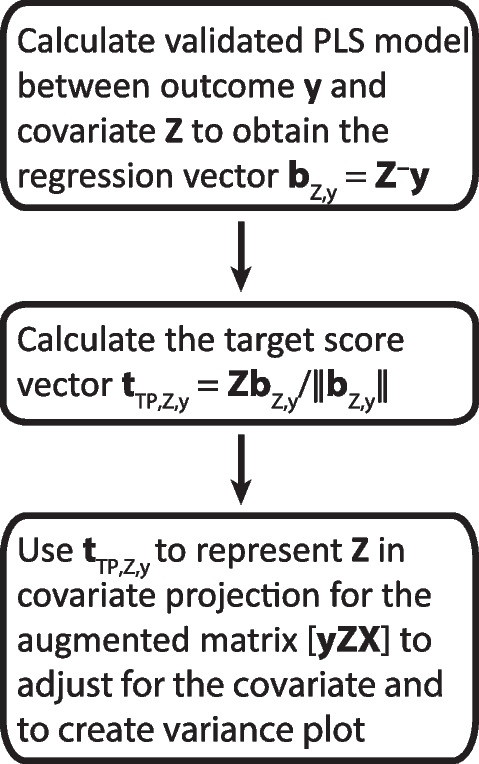
Flow diagram of covariate projection for a multicollinear covariate, i.e., the mediating lipoproteins in this work. Note that the projection step includes the mediator. This is necessary to be able to calculate the variance pattern of the mediator. The variance pattern of all covariates is displayed in variance plots which are crucial for interpretation

Figure 2 shows the procedure for a single multicollinear covariate. The mediator in this work represents an example of such a covariate. The method can handle more complex situations with several and different kind of covariates as illustrated in our application where covariate projections are performed in a stepwise manner: First, for the confounders age and sex represented by single variables, and, subsequently, for the multicollinear mediating lipoprotein profile represented by the target component score. Note that it is not mathematically necessary to adjust the explanatory variables for the mediator to obtain the relation of the net explanatory to the net outcome. By adjusting the outcome for the mediator, we have already removed the possibility of this part of the associations to influence the model after adjustment. We adjust the explanatory variables in our implementation for two reasons: i) to obtain a general algorithm for covariate projection and, ii) to obtain a variance plot that includes the variance pattern relating the mediator to the explanatory variables as it does for confounders and potentially other covariates.

### Total model and variance plot

As shown by Eq. 3a, our approach to adjust for covariates, separates the matrix ***X*** into orthogonal parts within the frame of a single model. After postprocessing the PLS model relating ***y***_*net*_ to ***X***_*net*_ using repeated Monte Carlo resampling by target projection, we can rewrite the total model (Eq. 3b) for ***X*** as

5a $${\varvec{X}}= {\widehat{{\varvec{X}}}}_{cov}+{\widehat{{\varvec{X}}}}_{net,TP}+{{\varvec{E}}}_{X,TP}={\sum }_{a=1}^{{n}_{CP}}{\widehat{{\varvec{X}}}}_{cov,a}+ {{\varvec{t}}}_{net,TP}{{\varvec{p}}}_{net,TP}^{T}+ {{\varvec{E}}}_{X,TP}$$

where *n*_*CP*_ is the number of covariate projections performed.

Thus, we first use the projection algorithm to isolate the part of ***X*** related to the covariates in the matrix, ${\widehat{{\varvec{X}}}}_{cov}$, and second, we calculate a validated PLS model for the associations between the adjusted outcome and explanatory variables that is postprocessed by target projection using the weight vector ***w***_*net,TP*_ = ***b***_*ne*t_/‖***b***_*ne*t_‖ to obtain the contribution ***X***_*net,TP*_ related to the adjusted outcome. Thus, the total model partitions the variance of ***X*** into a description of the covariates, the adjusted *predictive* part of ***X*** associated with the outcome, and a residual matrix ***E***_*X,TP*_. The partition of ***X*** provides the basis for model visualization in a variance plot [11].

It is possible to further partition the variance of the residual matrix using, e.g., PCA, to reveal association patterns unrelated to the outcome [16].

The outcome is similarly partitioned as

5b $${\varvec{y}}= {\sum }_{a=1}^{{n}_{CP}}{\widehat{{\varvec{y}}}}_{cov,a}+ {\widehat{{\varvec{y}}}}_{net}+ {{\varvec{e}}}_{y}$$

### Visualization of variance patterns

Variance patterns displaying how outcome and explanatory variables relate to covariates and to each other can be calculated from Eqs. 5a and 5b and visualized in variance plots. For the application in this work, the variance is decomposed as

6a $${\varvec{X}}= {\widehat{{\varvec{X}}}}_{Age}+ {\widehat{{\varvec{X}}}}_{Sex}+ {\widehat{{\varvec{X}}}}_{Lipoproteins}+ {\widehat{{\varvec{X}}}}_{net,TP} + {{\varvec{E}}}_{X,TP}$$

6b $${\varvec{y}}= {\widehat{{\varvec{y}}}}_{Age}+ {\widehat{{\varvec{y}}}}_{Sex}+ {\widehat{{\varvec{y}}}}_{Lipoproteins}+ {\widehat{{\varvec{y}}}}_{net}+ {{\varvec{e}}}_{y}$$

The interpretability of variance plots is improved by including covariates in addition to outcome and explanatory variables.

For the study of variance patterns and variable importance in models, we have developed several measures [17]. The choice of measure depends on the objectives of the application. Here, we want to see the changes in pattern accompanying confounder projections and a mediator projection. The best measure for this purpose is the selectivity fraction (SF) plot:

7 $$SF_{i} = \left\| {t_{TP} p_{i,TP} } \right\|^{2} /\left\| {x_{i} } \right\|^{2} \;\;\;\{ i = 1,2, \ldots ,M\}$$

The only difference between SF and the more commonly used selectivity ratio (SR) is the division by total variance ‖***x***_*i*_‖^*2*^ instead of the residual variance ‖***e***_*i,TP*_‖^*2*^. By relating variable importance to the fraction of total variance, we obtain a measure of explained *predictive* variance for the explanatory variables varying between − 1 and + 1. We use Eq. 7 for both unadjusted and adjusted data in this work.

Other options are available for interpretation of patterns and variable importance in models. Comparative studies performed by Farres et al. [18] and Mehmood et al. [19] confirm the usefulness of the selectivity ratio and thus the related measure selectivity fractions. A comparison with other available measures is outside the scope of this work.

### Software description

The projection algorithm for the various steps was implemented in an open-source R package called mvpa. The software is available on GitHub (github.com/liningtonlab/mvpa). To facilitate data handling and processing, we integrated the mvpa R package into an R shiny graphical user interface called mvpaShiny (github.com/liningtonlab/mvpaShiny). The packages were designed to be a broadly applicable toolbox for multivariate datasets, allowing stepwise adjustment for variables and analysis of associations. A detailed description of how to install and use the packages is available on the associated documentation page (https://liningtonlab.github.io/mvpaShiny_documentation). Both packages have been developed in R version 4.2 and make use of popular packages from tidyverse [20] for dataset handling and plotly [21] for interactive plotting. The basic PLS regression algorithm is from the chemometrics package of Filzmosers and Varmuza [22], but the validation of predictive PLS components uses the repeated Monte Carlo resampling algorithm of Kvalheim et al. [8]. For the generation of the shiny app, we used the packages shiny [23] and shinyjs [24]. For a detailed list of packages used and required versions, we refer users to the description file in the respective package repository.

#### Data import

Data may be imported in either.csv or.xls(x) formats. Data are validated to highlight columns containing missing values or invariant variables. Columns containing Boolean terms (yes/no, True/False) or strings may optionally be converted to numerical values.

#### Preprocessing

Data may be normalized, log transformed, standardized, min–max scaled, or transposed, either by column or by full dataset.

#### Subsetting

Data may be filtered by value ranges or subsets by the selection or exclusion of specific variables or objects.

#### Inspection

Variables can be inspected for normality via quantile–quantile plots and for correlation either against a single variable or as a correlation matrix.

#### Principal component analysis

Data can be subjected to PCA, optionally excluding specific variables and after adjustments using covariate projections. Outputs include scree, scores and loadings plots. In addition, a variable variation plot is provided that illustrates the contribution of each variable to each principal component. Optionally, the dataset may also be dimensionally reduced by selecting specific principal components.

#### Covariate projection

Covariate projections can be generated from selections of variables and are visualized in variance plots displaying variance patterns for each covariate projection.

#### PLS regression and target projection

PLS regression models can be generated using Monte Carlo resampling. User-modifiable variables include number components, number of repetitions, proportion of objects in calibration dataset and validation threshold. Additionally, users may select either RMSEP or MAE for the cost function. The results are displayed in a model information plot, permitting selection of the number of retained components. Target projection is performed automatically, and the resulting target loading vector may be further processed and displayed in bar plots, such as selectivity ratio or selectivity fraction plots. The results are also displayed as a variable variance distribution plot based on the target projection.

At all stages in the processing pipeline, datasets may be visualized and saved. In addition, all plots are interactive and may be scaled, zoomed, and saved as images.

The online documentation describes the steps for either the R version or the mvpaShiny app to reproduce the results of the application example described in the next section.

### Application example

**Fig. 3:**
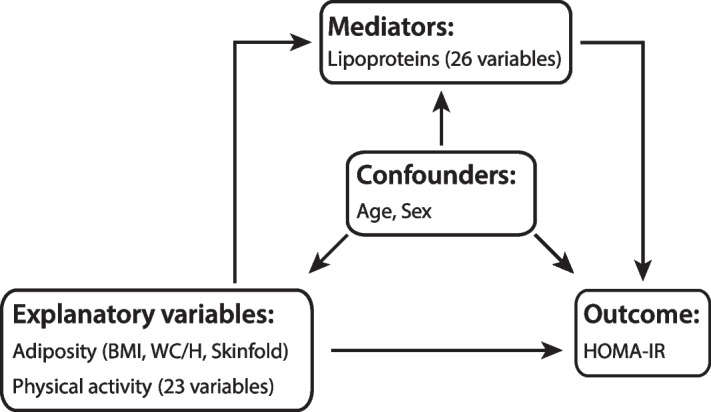
The model structure displaying the relations between groups of variables. HOMA-IR, which is a proxy for insulin resistance, is predicted from two groups of explanatory variables, i.e., three measures of adiposity and a physical activity descriptor providing the number of counts at 23 intensity intervals derived from an accelerometric sensor. Age and sex are confounders, and a profile of 26 lipoprotein measures acts as a mediator between HOMA-IR and adiposity and physical activity. Arrows imply the directions of the relations

It is well known that adiposity (obesity) and physical inactivity promote insulin resistance and that there is a strong association of the serum lipoprotein profile with both these lifestyle-related factors and insulin resistance [13] and references therein. The aim of the worked application is i) to show how the projection algorithm can be used to adjust for confounders and quantify the partial mediation of a comprehensive lipoprotein profile on the predictive association pattern between the homeostatic model assessment of insulin resistance (HOMA-IR) and adiposity and physical activity and ii) to show some of the most important plots for model visualization and interpretation implemented as part of the software. Figure 3 displays the structure of the investigated model.

Figure 3 illustrates the relation between the variables. The net predictive associations between outcome and explanatory variables are predicted after adjustment for confounders and mediator as explained in the paragraph above accompanying Fig. 2. We use the projection method in all steps, but with projection criterion differing according to task.

### Description of data set

We used baseline anthropometrics, metabolomics, and physical activity data for 836 subjects from the active smarter kids (ASK) study [25]. Since procedures to obtain the data are thoroughly described in previous investigations [7, 11, 12], only a brief description is provided here.

### Outcome: homeostatic model assessment of insulin resistance (HOMA-IR)

HOMA-IR was calculated as fasting serum insulin times fasting serum glucose divided by 22.5 [26]. The product of fasting plasma insulin of 5 μU/ml and normal fasting plasma glucose of 4.5 mmol/l is 22.5. This value represents an individual with “normal” insulin sensitivity and a HOMA-IR score equal to 1 [27].

### Explanatory variables: physical activity spectrum and adiposity

Physical activity (PA) data were obtained using the ActiGraph GT3X + accelerometer [28] worn at the waist over seven consecutive days, except during water activities (swimming, showering) or while sleeping. We use a PA descriptor of 23 intervals covering the intensity spectrum of the vertical axis [7]. The intervals used for the descriptor were 0–99, 100–249, 250–499, 500–999, 1000–1499, 1500–1999, 2000–2499, 2500–2999, 3000–3499, 3500–3999, 4000–4499, 4500–4999, 5000–5499, 5500–5999, 6000–6499, 6500–6999, 7000–7499, 7500–7999, 8000–8499, 8500–8999, 9000–9499, 9500–9999 and ≥ 10,000 counts per minute (cpm). Time (min/day) spent in each of the PA intensities was calculated for the children.

We used three measures of adiposity: body mass index (BMI) (kg/m^2^), the ratio of waist circumference to height (WC/H), and skinfold thickness (cm) derived from measurements at four places (biceps, triceps, subscapular, and suprailiac).

### Confounders: age and sex

Age is measured as a continuous variable. The subjects were all 5th graders, so the age range was narrow. Sex is included as a binary variable, 0 for girls and 1 for boys, to be able to make one joint model incorporating both sexes.

### Mediator: lipoprotein features

Serum lipoprotein profiles were characterized by 26 variables predicted from proton nuclear magnetic resonance spectra with chromatographic measurements as reference values [11, 12]: Concentrations of total cholesterol (TC), triglyceride (TG), chylomicrons (CM), very-low-density lipoproteins (VLDL), low-density lipoproteins (LDL), high-density lipoproteins (HDL), two subclasses of CM (CM-1 and CM-2), five subclasses of VLDL (VLDL-L1, VLDL-L2, VLDL-L3, VLDL-M, VLDL-S), four subclasses of LDL (LDL-L, LDL-M, LDL-S, LDL-VS), six subclasses of HDL (HDL-VL1, HDL-VL2, HDL-L, HDL-M, HDL-S and HDL-VS), and the average particle size of VLDL, LDL and HDL subclasses.

### Transformations and pretreatment of variables

It is not a necessary assumption that the variables are normally distributed for the application of the methods implemented in the MVPA package, but the Monte Carlo resampling method used to validate the number of PLS components produces more stable results if the variables are approximately normally distributed. All variables, except age and sex, were therefore log-transformed. Prior to the statistical analysis, the data were mean-centered and standardized to unit variance.

### Target component model for the association of HOMA-IR with lipoproteins

**Fig. 4:**
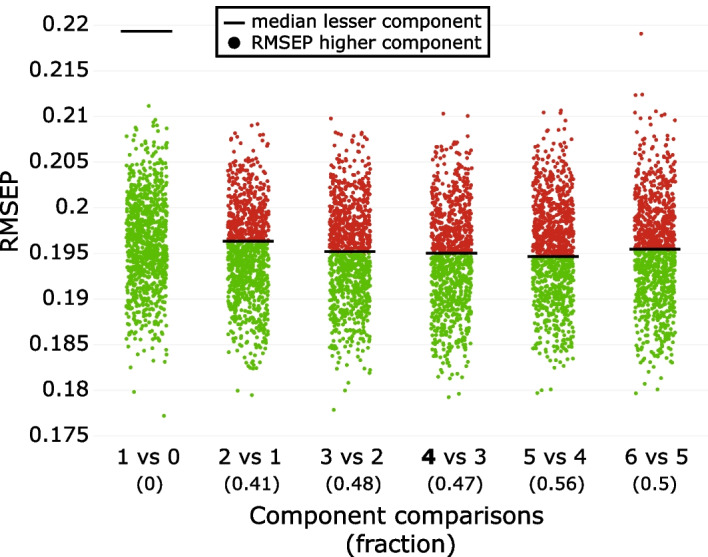
Validation plot to decide the number of predictive PLS components for the association pattern of the mediating lipoproteins to the outcome HOMA-IR. The plot displays the median value (black line) for Monte Carlo resampling with 1000 repetitions for an increasing number of components (starting from 0 and ending with 6 components which was chosen as the maximum number of components). The minimum median for RMSEP is found for the 4-component PLS model (indicated by *), implying that the number of predictive PLS components is lower than or equal to 4. The plot further compares the distribution of RMSEP values for the *A*-component models with the median of the (*A*-*1*)-component models. Red dots imply RMSEPs for the *A*-component models that exceed the median for the (*A*-*1*)-component model, while green dots indicate lower RMSEPs for the *A*-component models compared to the median of the (*A*-*1*)-component model. The numbers in parentheses show the fraction of the *A*-component model with RMSEPs exceeding the median for the (*A-1*)-component model. We start our assessment by comparing the 4-component model (minimum median of RMSEP) with the 3-componen model. The number 0.47 represents the fraction of repetitions for the 4-component PLS model, which exceeds the median for the 3-component model. Since the ratio of objects to variables is high in our data, we used 0.5 as the acceptance threshold. If this number is low the chance of overfitting increases and a lower number is recommended [8]. Thus, the 4-component model was chosen

PLS regression was performed for the unadjusted data with Monte Carlo resampling repeated 1000 times using 50% of the samples as calibration samples randomly selected and the other 50% as prediction samples. This procedure showed that 4 PLS components carried predictive information about the associations between HOMA-IR and the 26 lipoprotein variables (Fig. 4).

The validation plot shows that the minimum median for RMSEP is obtained for a 4-component PLS model. Furthermore, only 47% of the RMSEPs for the 4-component PLS models exceed the median RMSEP for the 3-component PLS model, further confirming that the 4-component model has the best predictive performance. This PLS model explained 24.4% of the variance in HOMA-IR. Postprocessing this model by performing a target projection provided the predictive lipoprotein pattern associated with HOMA-IR. This target component explained 32.7% of the total variance in the lipoproteins. The standardized target component score vector is subsequently used for adjustment in the projection algorithm to estimate the mediation effect of the lipoproteins on the physical activity spectrum and the three adiposity measures in their associations with HOMA-IR.

To validate that all predictive information had been extracted by the target lipoprotein component, regression of the lipoproteins on HOMA-IR after adjusting the data for age, sex and the target lipoprotein component was performed. Repeated Monte Carlo calculations confirmed that predictive associations between HOMA-IR and lipoproteins were exhausted by the target model (see Fig. 7 below).

### Correlation between covariates

Before discussing association patterns after adjustment for the confounders age and sex and the mediating lipoproteins as represented by the target component, we explore the correlations of these covariates to the outcome HOMA-IR. The results are shown in Table 1.

**Table 1:** Correlation coefficients between the covariates and HOMA-IR

	Age	Sex	Lipoproteins	HOMA-IR
Age	1	− 0.012	− 0.054	0.033
Sex		1	0.162	0.176
Lipoproteins			1	0.494
HOMA-IR				1

The confounders age and sex are almost uncorrelated to each other. Age is also almost uncorrelated to HOMA-IR and to lipoproteins, while sex possesses a moderate correlation to both HOMA-IR and lipoproteins. The strongest correlation is observed between HOMA-IR and lipoproteins.

### Variance reduction in data and models caused by covariate projections

Covariate projections were performed in the following order: First, for the confounders age, sex, and. then, for the mediating lipoprotein target component. The same Monte Carlo validation procedure used for modelling the predictive association of HOMA-IR with lipoproteins produced predictive models with 7 PLS components for the association of HOMA-IR with adiposity and physical activity for unadjusted as well as for the various adjusted versions of the data.

The three first columns of Table 2, with headings marked with superscript a, list the remaining variance in percent of original variance for the outcome (HOMA-IR) and the two groups of explanatory variables (the three adiposity variables and the 23 PA variables) for unadjusted data and after stepwise covariate projections in the order age, sex, and lipoproteins. The last three columns, with headings marked with superscript b, show the variances explained as percent of the original total variance (variance before any adjustments) for the corresponding target component models between HOMA-IR and adiposity and PA.

**Table 2:** Remaining^a^ and explained^b^ variances of corresponding models for HOMA-IR, adiposity, and the PA spectrum before and after covariate projections

Adjusted for	V(HOMA-IR)^a^	V(Adiposity)^a^	V(PA)^a^	V(HOMA-IR)^b^	V(Adiposity)^b^	V(PA)^b^
Unadjusted	100	100	100	30.3	72.2	16.4
Age	99.9	99.9	99.8	30.2	72.4	16.4
Age and sex	96.8	95.9	95.8	27.2	73.6	13.2
Age, sex, and lipoproteins	74.3	81.1	92.0	13.1	54.1	7.5

We observe from Table 2 that adjustment for age has almost no effect on the variances of outcome and explanatory variables. This is not surprising since age for the subjects spans a narrow interval in the analyzed cohort, i.e., 10.22 ± 0.29 years. The explained variances in the model were also barely changed after adjustment for age. Adjusting additionally for the confounder sex provides a few percentage reductions in variances for the outcome and the explanatory variables. This is accompanied by a reduction in the explained variances in HOMA-IR and PA in the corresponding model, while the variance explained in adiposity is almost unchanged. This is not surprising since previous investigations have established that girls are less physically active than boys for this cohort [11, 12] and thus also have higher HOMA-IR. However, adjusting for the lipoproteins using the target component profoundly changes explained variances for the model; particularly for the outcome HOMA-IR, which is more than halved, but also for PA, which is reduced from 13.2% to 7.5%, a relative decrease of 43%, and, although to a lesser degree, for adiposity, which experiences a relative decrease of 26%. These observations confirm the partial mediation of lipoproteins on the association between HOMA-IR and lifestyle-related factors.

### Patterns explained and visualized in variance plots

**Fig. 5:**
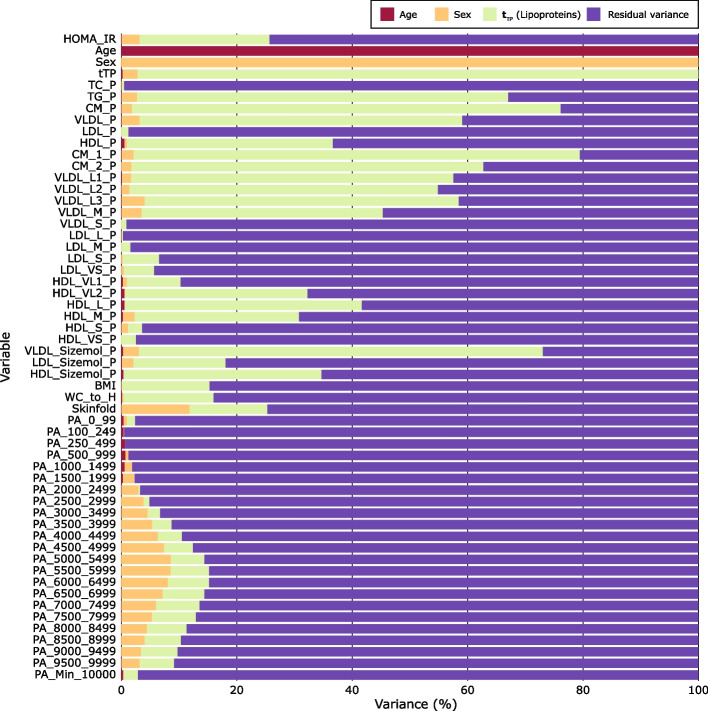
Variance pattern with adjustment performed in the following order: age, sex, and lipoprotein target component representing the predictive association pattern with HOMA-IR

We can quantify and visualize the influence of the covariate projections on the association pattern in a variance plot. Figure 5 displays the variance pattern of all variables resulting from the covariate projections performed in the same order as in Table 2, i.e., first the confounders age and sex, then the mediating lipoproteins represented by the target component obtained from the modelling of the predictive association of HOMA-IR to the 26 lipoprotein features.

Some observations from the variance plot are as follows: (i) Age shares almost no variance with any other variables, reflecting that age is almost uncorrelated to all the other variables in the narrow age range for this cohort. (ii) Sex associates moderately with the adiposity measure skinfold (explaining 11.9% of the total variance) and with increasing PA intensity, with a maximum correlation in the intensity region of 5–6000 cpm (explaining 8.6% of the original variance) but less with HOMA-IR (explaining 3.2% of the total original variance). These observations reflect that girls in our cohort were more prone to an increase in skinfold and spent less time in PA than boys [11, 12]. (iii) The mediating lipoproteins represented by the target score are moderately associated with all the adiposity measures (explained variances 11–13%) and with moderate- and high-intensity PA (5–7% of the total variance of each PA variable in the interval 4500–10000 cpm is explained by the lipoprotein target score).

The predictive explained variances for adiposity and PA, the last two columns in Table 2, show that the mediation effect of lipoproteins on HOMA-IR is relatively stronger for PA than for adiposity, but PA and adiposity are not independent of each other. Thus, previous work [11, 12] revealed a moderate inverse relationship between adiposity and PA.

### Model visualization and interpretation

**Fig. 6:**
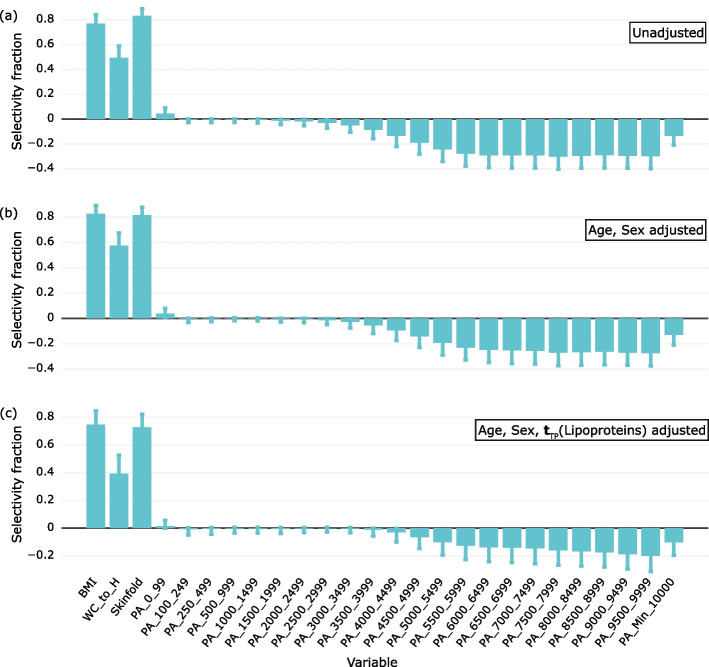
The association patterns of HOMA-IR with adiposity and physical activity with **a** unadjusted data, **b** data adjusted for the confounders age and sex, and **c** data adjusted for age, sex and the lipoprotein target component associated with HOMA-IR. Medians and 2.5 and 97.5% confidence limits are derived from the RMSEP distributions of 1000 models calculated by repeated Monte Carlo resampling

For model interpretation, visualization of the association pattern and ranking of the exploratory variables according to their importance for predicting the outcome are crucial. The MVPA package contains several tools for this task [17]. In this work, we use the selectivity fraction (SF) plot for this purpose. The SF plot displays the fraction of total variance explained by the target component for each exploratory variable. We use SF plots to examine how confounders and mediators impact the interpretation of the association pattern between HOMA-IR and adiposity and PA. Figure 6 shows the patterns for unadjusted and adjusted data:

Adjustment for the confounders age and sex (Fig. 6b) shows a weakening of the association of the PA spectrum with HOMA-IR, while the association with the adiposity measures is unchanged. This conforms with the results in Table 2. The variance explained for the model after adjusting for sex shows a reduction in predictive PA variance from 16.4 to 13.2%. This observation is ascribed to less time spent on PA among girls in our cohort compared to boys.

Adjustment for lipoproteins causes a greater change in the association pattern (Fig. 6c): The association between HOMA-IR and adiposity as measured by WC/H is strongly reduced. This adiposity measure is a proxy for abdominal fat, which is associated with HOMA-IR [29]. Furthermore, the association with the PA spectrum is almost halved, while the variance explained in HOMA-IR is more than halved. Thus, lipoproteins have a partial mediating effect on the association pattern of HOMA-IR with lifestyle-related factors.

### Influence of residual variance in the lipoproteins on model interpretation

The univariate confounders age and sex had zero variance after adjustment by covariate projections and only marginally influenced the variances in the 26 lipoprotein features (1.4% shared variance). However, a large amount of residual variance is present in the lipoprotein measures after the covariate projection since the lipoprotein target component used for the projection only explained 32.7% of the original variance in the lipoproteins. The large residual variance in lipoproteins may suggest potential problems for the interpretation of the net regression model relating HOMA-IR to adiposity and PA. However, since our targeted approach adjusts for the systematic predictive variance of the lipoprotein associated with HOMA-IR, confounding due to residual variance is in fact shown to be of no concern.

To quantify the possible impact of residual confounding caused by adjusting for the multivariate mediator by projecting the variables on the lipoprotein target score, we modelled HOMA-IR with the lipoproteins included as explanatory variables together with adiposity and PA for the data after adjustment for sex, age, and the lipoprotein target score. Inclusion of the adjusted lipoproteins adds noise to the model, so a 3-component PLS model is predictive, explaining 12.7% of the original variance in HOMA-IR, which can be compared with 13.1% for the 7-component model without the lipoproteins.

**Fig. 7:**
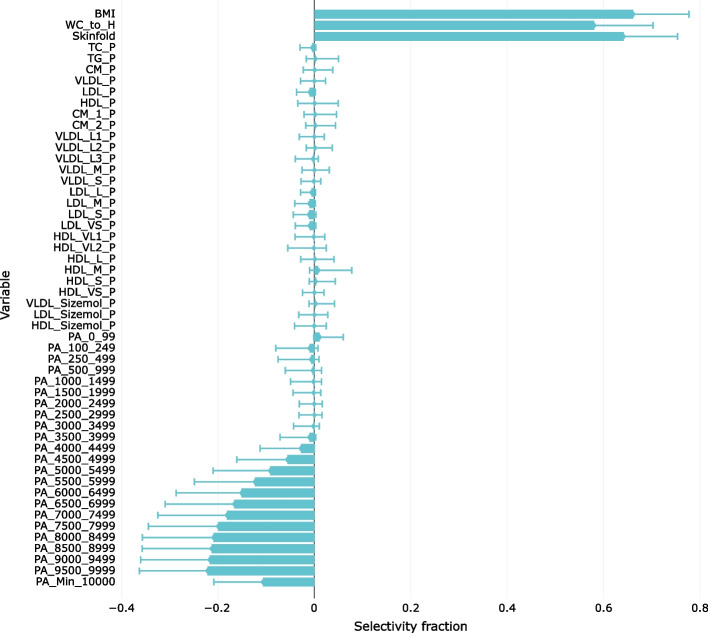
The association patterns with data adjusted for age, sex and the lipoprotein target component associated with HOMA-IR and with the 26 lipoprotein features included as explanatory variables. Medians and 2.5 and 97.5% confidence limits are derived from the RMSEP distributions of 1000 models calculated by repeated Monte Carlo resampling

The associations between HOMA-IR and the explanatory variables are displayed in the selectivity fraction plot (Fig. 7).

Evidently, the SF plot displays no significant associations with the lipoprotein measures. Only 0.4% of the original total variance of the 26 lipoprotein features is accounted for by the model. The explained variances in the PA and adiposity variables are 8.4 and 52.5%, respectively, which is in line with the result for the corresponding model not including the lipoprotein variables (Table 2). However, the reduction in association between HOMA-IR and the adiposity measure WC/H is lost in the lower-dimensional PLS model due to the noise introduced by adding the nonrelevant adjusted lipoproteins, which leads to a lower-dimensional PLS model.

## Discussion

MVPA for handling multicollinearity in covariates and explanatory variables by projection methods is implemented in R as an open-source package. The software includes a graphical user interface to determine, visualize and interpret association patterns. Repeated Monte-Carlo resampling is used to determine the number of PLS components to be included in the target projection postprocessing step providing the predictive association pattern.

The projection algorithm provides variance patterns for covariates as an integrated part of the model. Taking covariates into account is crucial in analysis to arrive at estimates of independent associations between explanatory and outcome variables. In the first applications of MVPA, adjustment was performed using residualized variables from MLR [6, 7]. However, this process is tedious with many explanatory variables and thus does not work when covariates are linearly dependent. In two recent applications [11, 12], we used PCA to handle linearly dependent covariate. A drawback with this procedure is that many principal components are usually necessary to describe multivariate covariates. This complicates visualization and interpretation. This is resolved by condensing the associations of a multivariate covariate to the outcome and the explanatory variables on a single target component. Handling multicollinear covariates in the way exemplified by the lipoprotein profiles in this work, and including them as an integrated part of the model is therefore an important step towards an effective, streamlined approach for analyzing complex, multicollinear datasets. In addition to efficiently adjusting for covariates, we can quantify and visualize this part of the total model using variance plots. The approach is transparent and works stepwise, which makes it easy to observe problems caused by collinearity. Importantly, the implemented method handles linearly dependent covariates, which is common in metabolomics applications.

The influence of the covariates can be quantified as their cumulative part of the total variance in unadjusted data as well as for each variable individually in variance plots. This allows for assessing the influence of covariates on the model and the individual variables constituting the model.

Equations 5a and 5b imply stepwise projection, i.e., one projection for each single or multivariate covariate. This approach has the advantage that it provides possibilities for detailed visualization and interpretation of covariates since it partitions the variance into parts relating covariates to specific association patterns. This is the approach we use in the application in this work. However, if some or all the covariates are strongly associated, a single projection including all the strongly associated covariates may be preferable, as implied by Eqs. 4a and 4b. Covariate patterns are orthogonal to the remaining variance pattern so that the variance pattern of the net data can be analyzed and interpreted independently of their associations to the covariates.

The software includes excellent possibilities for visualization and interpretation. Ranking of variable importance is available as SR and SF plots. However, additional tools are available for exploring predictive correlation patterns and quantifying variable importance, namely, the so-called multivariate correlation coefficient plot and the multivariate covariance coefficient plot [17]. While SR and SF plots are built from and related directly to the predictive model, the multivariate correlation (standardized) and covariance (unstandardized) coefficient plots take the explained variance of the PLS model into account and can be interpreted as equivalent to bivariate correlation or regression coefficients, respectively, except that they are derived from and must be interpreted in the multivariate space. Thus, the coefficients relate directly to the actual outcome, which substantially eases the comparison of association patterns across, for example, models for different outcomes or different groups. While recommending the multivariate correlation coefficient on this background, we urge researchers to exercise caution in using the multivariate covariance coefficient for strongly multicollinear explanatory variables since the variables carry (more or less) the same information but with potentially very different standard deviations. For instance, different choices of binning of variables may have a great impact on these covariance coefficients. This may substantially complicate interpretation, particularly when there is a lack of a standard operationalization of the explanatory variables. Furthermore, it may mislead researchers to interpret associations as independent, which they are not.

The developed software provides a tool with broad applications. Most data produced by sensors and instruments are inherently multicollinear, and it is advantageous to take full advantage of the high resolution usually delivered instead of reducing the resolution to derive data that fit the assumption of less general analytical methods. There are no limitations on the number of explanatory variables or covariates that the mvpa software can handle. However, the method and the software can presently only handle one outcome at a time.

It is also an advantage that all the methods needed to analyze and visualize the data can be included in a common mathematical frame based on the projection algorithm. Only the criteria are different depending on whether covariates are univariate or multivariate and possibly linearly dependent.

While there are many software packages that can analyze data with multicollinear data, there is no software package available, to our knowledge, that can handle linear dependent covariates such as the serum lipoprotein profile mediating the relation between the explanatory variables and the outcome in the application investigated in our work and provide the type of model interpretation and visualization presented here.

## Conclusion

MVPA represents a tool for studying and visualizing association patterns in complex, multicollinear data. The implemented software in R handles situations with multicollinear covariates that influence association patterns in regression models. The method works irrespective of the number of covariates and for linear dependent covariates and explanatory variables. Furthermore, the method treats covariates as an integrated part of the model and acknowledges the complementary and important information supplied by these variables. Interpretation of their variance pattern shared with outcome and explanatory variables may provide additional insight into important aspects of the data.

### Supplementary Information

**Additional file 1.** Data analyzed in this work after preprocessing but prior to any adjustments.

## Data Availability

Project name: Multivariate Pattern Analysis R shiny package. Project home page: https://github.com/liningtonlab/mvpa, https://github.com/liningtonlab/mvpashiny. Operating system(s): Windows, MacOS, Linux. Programming language: R. Other requirements: RStudio for mvpaShiny. License: GPL-3.0 license. Any restrictions to use by non-academics: None. The active smarter kids (ASK) study is registered in Clinicaltrials.gov with identification number NCT02132494. The baseline data from the ASK study analyzed in this study are included in this article as supplementary information (Additional file 1).
